# How Action Shapes Temporal Judgments: A Study in Brain Damaged Patients Through Immersive Virtual Reality

**DOI:** 10.3390/jcm14144825

**Published:** 2025-07-08

**Authors:** Greta Vianello, Michela Candini, Giuliana Vezzadini, Valentina Varalta, Gennaro Ruggiero, Tina Iachini, Francesca Frassinetti

**Affiliations:** 1 Neurorehabilitation of the Institute of Castel Goffredo, Istituti Clinici Scientifici Maugeri IRCCS, 46042 Castel Goffredo, Italy; greta.vianello@gmail.com (G.V.); giuliana.vezzadini@icsmaugeri.it (G.V.);; 2 Department of Psychology “Renzo Canestrari”, University of Bologna, 40126 Bologna, Italy; 3 Neuromotor and Cognitive Rehabilitation Research Center, Section of Physical and Rehabilitation Medicine, Department of Neurosciences, Biomedicine and Movement Sciences, University of Verona, 37129 Verona, Italy; valentina.varalta@univr.it; 4 Neurorehabilitation Unit, University Hospital of Verona, 37126 Verona, Italy; 5 Laboratory of Cognitive Science and Immersive Virtual Reality, Department of Psychology, University of Campania “L. Vanvitelli”, 81100 Caserta, Italy; gennaro.ruggiero@unicampania.it (G.R.); santa.iachini@unicampania.it (T.I.)

**Keywords:** virtual reality, action, time estimation and reproduction, brain damaged patients, VLSM

## Abstract

**Background/Objectives**: Time processing is crucial for managing several aspects of our daily experiences: the continuous interaction with a changing environment requires individuals to make precise temporal judgments. Following right hemisphere damage, patients exhibited a significant alteration in perceiving temporal duration. However, this impairment usually emerges with “abstract” computerized tasks, not in everyday contexts. This study investigates estimation and reproduction of time intervals in left (LBD) and right brain damaged (RBD) patients compared to healthy controls. **Methods**: We adopt computerized tasks (Experiment 1) and novel virtual reality (VR) tasks where participants judged the duration of their own actions framed within a realistic VR context (Experiment 2). **Results**: RBD but not LBD patients underestimated time intervals, and reproduced time intervals as longer than they are. Crucially, when participants judged the temporal duration of meaningful actions performed in a realistic context through the VR scenarios, the impairment in processing time observed in RBD patients was reduced. The Voxel-lesion-symptom-mapping (VLSM) analysis revealed the neurocognitive basis of time perception. **Conclusions**: Our results show that meaningful actions within familiar contexts can provide a channel of information that is essential for optimal time processing, suggesting the importance of assessing time processing in an ecologically controlled manner using VR.

## 1. Introduction

The temporal dimension is one of the most distinctive aspects of our daily experiences: our actions unfold over time and rely on accurate timing [1,2]. Our brain actively and continuously builds the temporal dimension, considering both external and internal events [3]. For instance, detecting deviations in a temporal rhythm requires processing time as an intrinsic and exogenous property and mainly depends on externally cued timing processes. Conversely, internally based timing mechanisms are grounded on our inner sense of the flow of time, independent of contextual cues: discriminating between two duration intervals (time estimation task), or pressing a key when a defined temporal interval is thought to have elapsed (time reproduction task) are examples of internally based timing processes [4,5].

In this respect, the neuropsychological literature has documented an altered sense of temporal flow in patients following a focal brain lesion: indeed, time perception seems to be selectively impaired in right brain damaged (RBD) patients, who underestimate time durations and over-reproduce temporal intervals, whereas no such temporal deficit has been found following left brain damage (LBD) [6,7,8,9,10,11,12]. In these studies, participants were required to judge the duration of a visual static stimulus presented on the computer screen. However, previous evidence on healthy participants demonstrated that the perception of moving stimulus modulates temporal judgments too [13,14,15,16,17,18]. In this respect, Giorjiani and colleagues [19] showed that temporal judgments of moving stimuli (i.e., body-parts) changed if the stimulus moved according to a biological or non-biological movement, that is, or not plausible with the human motor repertoire. Recently, Pacella and colleagues [20], explored the role of biological or non-biological movements in temporal judgments in a group of left and right brain damaged patients. Results showed that LBD but not RBD patients failed in temporal estimation of biological movements, whereas a slowdown of temporal judgments in RBD patients was found when judging the non-biological movements as compared to controls. Thus, both the dynamicity and biological plausibility of a stimulus influence temporal processing. These findings are coherent with the embodied nature of temporal processing, grounded on the close relationship between action and perception of durations, and highlight the role of one’s own action in temporal processing [21].

Moreover, it is well known that temporal disorders are often revealed in clinical contexts but are rarely reported by patients in ecological contexts. To date, indeed, most of the studies have investigated temporal deficits through computerized tasks, which are not representative of everyday life. This raises the question of whether it is possible to detect these disorders in everyday-like contexts while maintaining the necessary control over the variables of interest. Thus, we developed a novel paradigm based on Virtual Reality (VR) technique. VR seems a natural candidate for this work since it allows keeping under control the spurious variables that inevitably characterize complex real-life contexts and can undermine the sensitivity of the devised paradigm [22,23,24]. Therefore, VR makes it possible to develop familiar tasks in ecologically valid contexts that maintain the expectations of corresponding real-life situations, ensuring a high degree of experimental control and ecological validity [25,26,27].

Our general hypothesis is that temporal deficits should be confirmed in RBD patients on computerized tasks, but should be attenuated when they judge the duration of actions performed in everyday life. The effect could be different when estimation rather than reproduction of action duration is required.

To test our hypotheses, RBD and LBD patients and a group of age-matched healthy controls were submitted to two experiments assessing time estimation and time reproduction. In Experiment 1, two computerized tasks were adopted, whereas in Experiment 2, a novel ecological paradigm to assess time through VR was introduced. In Experiment 1, participants were presented with a visual stimulus (i.e., a square) and they had to verbally judge each stimulus as “short” or “long” with respect to previously acquired pairs of reference durations (time estimation computerized task) or to reproduce the duration of the stimulus (time reproduction computerized task). In Experiment 2, participants were submitted to virtual scenarios (i.e., house and supermarket) where they performed everyday-life actions, and they had to estimate and reproduce the actions’ duration. In the time estimation Virtual Reality task, participants were required to verbally estimate the duration of an action before and after having performed it in the virtual scenario. In the time reproduction Virtual Reality task, participants had first to execute an action in the virtual scenario, and then they were required to reproduce the time it lasted.

Regarding computerized tasks, in agreement with the literature, we predict that RBD patients should exhibit a deficit both in time estimation and time reproduction tasks [11,13,28]. In particular, we expect a concurrent time underestimation and over-reproduction in RBD patients compared to LBD patients and healthy controls [6]. Accordingly to the Scalar Expectancy Theory (SET; [29]), the slowdown of the internal clock mechanism, observed in RBD patients, would lead to the underestimation of the passage of time while reproducing time intervals and, consequently, result in an over-reproduction.

Considering the VR tasks, estimation and reproduction of temporal durations of actions are embedded within everyday-life activities and realistic contexts. Notably, when participants carry out such tasks, a mental simulation of the motor sequence involved in the actions, even without physically performing the movement, is likely to be elicited, especially when people have to reproduce the action’s duration. Indeed, motor imagery is a form of motor simulation involving the internal generation of visual and kinesthetic aspects of movement in a first-person perspective and activates the motor system in the absence of motor execution [30,31]. Thanks to mental simulation of familiar and ecological actions, RBD patients’ temporal performance may be facilitated. If this is the case, this would mean that computerized and virtual tasks rely on distinct mechanisms and, consequently, they may be differently affected following a focal brain lesion. Indeed, while the right insula and the right post central gyrus have recently been pointed out as a core brain network for the ability to estimate and reproduce time duration of a static visual stimulus [6,32,33,34,35,36,37,38], estimating and reproducing actions’ duration might engage a brain network involved in the embodied cognition of time. It has been shown that mental imagery of body-parts (i.e., hands) relies on the activity of a bilateral cortical network that includes frontal, insular and temporo-parietal regions [39,40,41,42,43,44]. Therefore, we expect that providing a temporal judgment of a visual stimulus or of one’s own action may yield a dissociation in temporal processing between computerized and virtual reality tasks.

## 2. Materials and Methods

### 2.1. Participants

Thirty-four patients, 18 with right focal brain damage (RBD, 12 males, mean age ± SD 62.8 ± 10.5 years) and 16 with left focal brain damage (LBD, 9 males, mean age ± SD 64.3 ± 13.2 years), as well as 19 neurologically healthy controls (HC, 5 males, mean age ± SD 61.9 ± 7.5 years) were recruited in the study. Participants were matched for age [F_(2,50)_ = 0.221, *p* = 0.80, η^2^_p_ = 0.01] and education [F_(2,50)_ = 2.48, *p* = 0.09, η^2^_p_ = 0.09].

Patients with a left or right hemisphere stroke were consecutively recruited from the Service of Neuropsychology of the Istituti Clinici Scientifici Maugeri IRCCS (Castel Goffredo, Italy) and at the Department of Neuroscience, Biomedicine and Movement (Verona, Italy) between 2019 and 2023. Healthy participants were volunteers with no history of neurological or psychiatric disorder, recruited from the Confederazione Nazionale dell’Artigianato (CNA, Bologna, Italy). We determined the sample size of our study through the G∗Power software (v. 3.1.9.7). Based on the results of a previous study [6,10], we estimated a medium effect size n_p_^2^ = 0.17 for the main effect of Group (Critical F = 3.19), and set the significance level at α = 0.05 and the desired power (1 − β) at 0.95, leading to a minimum sample size = 51.

All subjects gave their informed consent for participation in the study, which was approved by the local Ethics Committee, and all procedures were performed in agreement with the World Medical Association Declaration of Helsinki (2013).

### 2.2. Neuropsychological Assessment

All participants were administered the Mini Mental State Examination (MMSE [45]; HC = mean score ± SD = 27.5 ± 2.2) as a measure of general cognitive functioning, and a questionnaire assessing cognitive estimation of time and weight (STEP [46]; HC = time subscale mean score ± SD = 23.9 ± 1.9; weight subscale mean score ± SD = 21.6 ± 3.8). Three One-way ANOVAs were conducted to compare the performance of the three groups at MMSE, and time and weight STEP subscales. No significant differences were found across groups (all ps > 0.15).

Patients were screened for eligibility to participate in the study and submitted to a more extended neuropsychological assessment. The following inclusion criteria were adopted: (1) presence of a unilateral hemisphere lesion confirmed by neuroradiological evaluation; (2) absence of verbal comprehension impairment (as assessed with Token Test [47]); (3) absence of visuo-spatial neglect (as assessed with Behavioural Inattentional Test (BIT—conventional subtests; [48]), Apple’s Test [49] and Bell Cancellation Test [50]). Patients were classified without neglect when reported scores were above the cut-off in at least two of the tests mentioned. When patients met the inclusion criteria, they were enrolled in the study. Moreover, executive and motor functions were assessed with the Frontal Assessment Battery (FAB; [51]). The motor subscale of the Functional Independence Measure (FIM; [52]) was also considered as an index of motor abilities in stroke patients (RBD = mean score ± SD = 53.8 ± 20.9; LBD = mean score ± SD = 64.8 ± 24.3) (see Table 1 and Table 2 for a detailed description of patients and controls, respectively).

### 2.3. Procedure

#### 2.3.1. Experiment 1

The Experiment 1 consisted of two computerized tasks that were presented in a counterbalanced order.

##### Time Estimation Computerized Task

**Figure 1:**
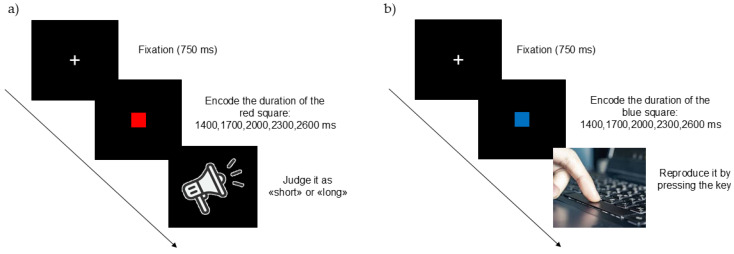
The experimental sequence for the Time Estimation (**a**) and the Time Reproduction (**b**) computerized tasks.

The time estimation computerized task consisted of the verbal classification of a series of visual stimuli (red squares) that were displayed for different durations at the centre of the computer screen. The visual stimulus durations were 1400, 1700, 2000, 2300, 2600 ms. Participants were instructed to verbally judge whether the duration of each square was “short” or “long” with respect to previously acquired pairs of reference durations (1400 ms and 2600 ms) (Figure 1a). We elected a verbal (as opposed to motor) response (as in [53,54]) to avoid the possible interference effects between spatial (left/right) positions and temporal (short/long) durations [55,56,57].

Before administering the experimental task, the practice session consisted of 10 trials and served to familiarize participants with the two reference durations (1400 and 2600 ms), randomly presented. Feedback on accuracy was given for practice trials only. After the experimenter had ensured that participants were confident with the practice session (80% of accuracy), 30 trials (6 for each time interval) were presented in random order.

##### Time Reproduction Computerized Task

In the time reproduction computerized task, the visual stimuli were blue squares (1 cm × 1 cm) presented at the centre of the computer screen (1400, 1700, 2000, 2300, 2600 ms). Next, a red square appeared on the screen and remained visible for as long as participants pressed the space bar on the keyboard. The task was to reproduce the entire duration of the preceding blue square (Figure 1b). Before starting the experimental session, participants practiced for 10 trials, and feedback on their performance was given. After the experimenter had ensured that participants were confident with the practice session, 30 trials (6 for each time interval) were presented in random order.

#### 2.3.2. Experiment 2

The actions used as stimuli in the virtual scenario have been chosen on the basis of a pilot study. Healthy participants were presented with sixteen actions in the virtual scenarios. Ten out of sixteen actions that required execution time between 1400 and 2600 ms (i.e., the temporal intervals corresponding to stimuli durations adopted in Experiment 1) were selected in Experiment 2.

The Experiment 2 consisted of two virtual reality tasks that were presented in a counterbalanced order.

##### Time Estimation Virtual Reality Task

The VR system consisted of a 3D computer-generated Immersive Virtual Environment displayed to participants through HMD (HTC Vive^TM^, HTC Corporation, Taoyuan, Taiwan). Participants sat in front of a VR computer monitor wearing the HMD that allowed them to see the virtual scenario at a distance of about 1.5 m from it. The video camera tracked both participants’ movements and position. Participants could freely explore different scenarios in a virtual house and a virtual supermarket and could interact with the items displayed by using the wand.

**Figure 2:**
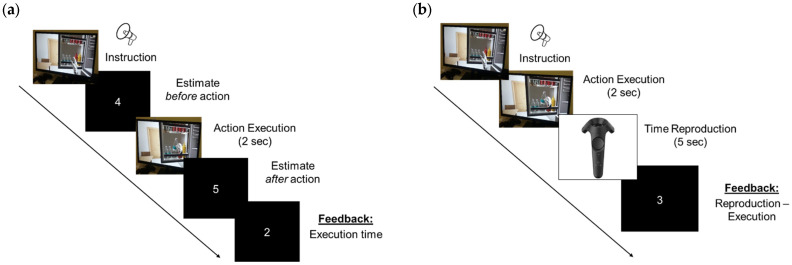
The experimental sequence for the Time Estimation (**a**) and the Time Reproduction (**b**) Virtual Reality task.

First, participants had to verbally estimate how long the action execution lasts (Estimation pre). Participants listened to a prerecorded voice instructing them to estimate the duration of an action congruent with the virtual context where they were immersed. For example, in the house, participants were required to take two bottles of water out of the fridge and put them on the table. Then, participants executed the same action by using the wand (Execution time) and finally, they were again required to verbally estimate how long the action execution lasted (Estimation post). For each action, a feedback was given about the execution time (expressed in seconds) (Figure 2a).

Ten real life actions were presented, five in the virtual house and five in the virtual supermarket (see Supplementary Material for a complete description of the 10 actions). A training session was conducted to familiarize with the task.

##### Time Reproduction Virtual Reality Task

Participants were submitted to the same VR system used in the previous experiment and the same ten actions, five in the virtual house and five in the virtual supermarket (see Supplementary Material).

In the Time Reproduction task, participants had first to perform an action by using the wand (i.e., Please, take two bottles of water out of the fridge and put them on the table) (Execution time). Then, participants were required to reproduce the duration of their performed action: to this end, they pressed the wand at the beginning and at the end of the estimated time action duration (Reproduction time). Feedback was given about the temporal difference between execution and reproduction (Reproduction time minus Execution time—expressed in seconds) (Figure 2b).

### 2.4. Data Analysis

To examine the impact of time deficits, four analyses of variance (ANOVAs) were separately conducted for computerized and VR tasks. In all analyses, *p* significance level was set at 0.05 and the confidence interval (CI) at 95% was calculated. Post-hoc analyses were conducted, where necessary, with the Honestly Significant Difference (HSD) test for Unequal N. Effect size is indicated as partial eta squared (η^2^_p_).

#### 2.4.1. Experiment 1

##### Time Estimation Computerized Task

The dependent measure was the mean proportion (in percentage) of “long” responses on the total number of trials. A percentage of “long” responses higher than 50% means that durations are perceived longer, reflecting a relative shift towards overestimation of temporal midpoint. A percentage of “long” responses lower than 50% means that durations are perceived shorter, reflecting a relative shift towards underestimation of temporal midpoint. For each participant, a Point of Subjective Estimation (PSE) was calculated as the stimulus duration to which the participants responded “short” or “long” with equal frequency. It is associated with the target duration corresponding to a predicted 50% rate of long responses: the smaller the PSE value, the longer the perceived duration. As a measure of time sensitivity, we also calculated, for each subject, the Weber Ratio (WR) as the standard deviation of the fitted cumulative curve (representing the proportion of “long” responses), divided by the PSE. Higher WR values are associated with a poorer time sensitivity.

Data on the mean proportion of “long” responses were analyzed using a two-way Analysis of Variance (ANOVA) with Group (RBD, LBD and HC) as a between-subject factor and Interval (1400, 1700, 2000, 2300, 2600) as a within-subject factor. A one-way ANOVA was also conducted on the PSE, taking Group (RBD, LBD and HC) as a between-subject factor. To compare time sensitivity across groups, the same one-way ANOVA was performed on WR.

##### Time Reproduction Computerized Task

The dependent measures were the mean reproduced interval (in milliseconds), the absolute error (AE), and the estimated-to-target duration ratio (Ratio). Reproduced time intervals longer than the encoded ones were interpreted as time underestimation, because subjects press the key later as if they believed that time is elapsing slower. Reproduced intervals shorter than the encoded ones were interpreted as time overestimation, because subjects press the key earlier as if they believed that time is elapsing faster. The AE was calculated as the difference between the time reproduction (R_d_) and the target duration (in absolute value; T_d_) divided by the target duration [AE = |R_d_ − T_d_|/T_d_] [13,58]. Large AE levels indicate low performance. The Ratio was obtained by dividing each participant’s time performance by the time duration of the interval presented for that trial [RATIO = R_d_/T_d_]. Coefficients above and below 1.0 were indicative of over-reproduction and under-reproduction, respectively.

Data on mean reproduced intervals were analyzed using an ANOVA with a two-way Group (RBD, LBD and HC) as a between-subject factor and Interval (1400, 1700, 2000, 2300, 2600) as a within-subject factor. Additionally, two separate one-way ANOVAs were conducted on Ratio and AE, taking Group (RBD, LBD and HC) as a between-group factor.

#### 2.4.2. Experiment 2

##### Time Estimation Virtual Reality Task

The dependent measures were Execution time of action (E_Est_), Estimation time measured before (Estimation pre) and after (Estimation post) action execution, and Difference (Estimation pre minus Estimation post) (the scores for each group are reported in Table 3).

Data on E_Est_ and Difference were analyzed using a series of ANOVAs with ***Group*** (RBD, LBD and HC) as a between subject-factor; data on Estimation time were analyzed by a two-way ANOVA with Group (RBD, LBD and HC) as a between-subject factor and Session (pre and post) as a within-subject factor.

##### Time Reproduction Virtual Reality Task

The dependent measures were Execution time of action (E_Repr_) and Reproduction time (the scores for each group are reported in Table 3). Data on E_Repr_ and Reproduction were analyzed using two one-way ANOVAs with **Group** (RBD, LBD and HC) as a between subject-factor.

#### 2.4.3. Regression Analyses

First, to verify whether estimation and reproduction time observed in VR were predicted by execution time, a series of regression analyses were conducted, separately for HC, RBD and LBD groups. In the Time Estimation virtual reality task, simple regressions were conducted on mean Estimation scores (pre and post) considering the E_Est_ as predictor. In the Time Reproduction virtual reality task, simple regressions were conducted on mean Reproduction score considering the E_Repr_ as predictor.

Second, to explore the relationship between the temporal performance on VR tasks and accuracy indices obtained in computerized tasks (Experiment 1), simple regression analyses were conducted, separately for HC, RBD and LBD groups. In the Time Estimation virtual reality task, a regression analysis was conducted on VR estimation scores (Estimation pre and post) considering the WR as predictor. Moreover, in the Time Reproduction virtual reality task a regression analysis was conducted on mean Reproduction scores considering the AE as predictor.

#### 2.4.4. Correlation Analyses

##### Computerized Tasks

First, to explore the relation between time underestimation and over-reproduction, Pearson’s correlation analyses were conducted considering the frequency of long responses (an index of Time estimation) and mean values of reproduction (an index of Time reproduction) in RBD and LBD patients.

Second, to verify the relationship between temporal ability measured in computerized tasks (i.e., PSE, WR and mean values of reproduction) and the neuropsychological performance, a series of Pearson’s correlation analyses were conducted on indices of spatial attention (i.e., overall score obtained at BIT-C), executive functions (i.e., overall score obtained at FAB, and the score obtained in the subscale of time and weight—STEP) and motor abilities (i.e., FIM), separately for RBD and LBD patients.

##### Virtual Reality Tasks

To investigate the relationship between the temporal ability in VR tasks and the neuropsychological performance in RBD and LBD patients, we conducted a series of Pearson’s correlation analyses considering the variables of estimation (E_Est,_ Estimation pre and post) and reproduction (E_Repr,_ Reproduction time) as indices of temporal ability. Moreover, indices of spatial attention (i.e., overall score obtained at BIT battery), executive functions (i.e., overall score obtained at FAB, and the score obtained in the subscale of time and weight—STEP) and motor abilities (i.e., FIM) were considered as neuropsychological performance.

### 2.5. Lesion Mapping

Brain lesions were identified by means of Computed Tomography and Magnetic Resonance digitalized images (CT/MRI) of 17 RBD patients and 14 LBD patients (3 out of 34 CT/MRI are missing due to technical reasons). For each patient, the location and extent of brain damage were delineated and manually mapped in the MNI stereotactic space by using MRIcro software [59]. First, to approximate the slice plane of the patient’s scan, the MNI template was rotated. Second, brain lesions were manually drawn (GV) onto each corresponding template slice by using anatomical landmarks. Then, drawn lesions were inspected by trained raters (FF and MC) and, in case of disagreement, an intersection lesion map was used. Finally, each lesion map was rotated back into the standard space, applying the inverse of the transformation parameters used in the stage of adaptation to the brain scan. The lesion overlay maps were superimposed on a ch2 template [60] using MRICro, separately for RBD and LBD patients.

Then, to understand which injured area was more associated with time underestimation and over-reproduction, a voxel-based lesion-symptom mapping (VLSM) method was used to correlate the anatomical extent of brain damage with the severity of temporal deficit, separately for the performance in time estimation tasks (computerized and virtual reality) and time reproduction tasks (computerized and virtual reality). We used the non-parametric Brunner-Munzel test [61,62] to perform statistical comparisons on voxel-wise bases, as implemented in the NPM and MRIcron software [63]. Brunner-Munzel tests were performed at each voxel using the temporal score as the dependent variable. The higher the resulting statistical output (Z value) relative to voxels in a given area, the stronger the association between damage in that area and impaired performance. In all analyses, permutation thresholding with 1000 iterations was used to apply corrections for multiple comparisons. The *p* significance level was set at 0.05 (Section 3.3).

## 3. Results

### 3.1. Experiment 1

#### 3.1.1. Time Estimation Computerized Task

**Figure 3:**
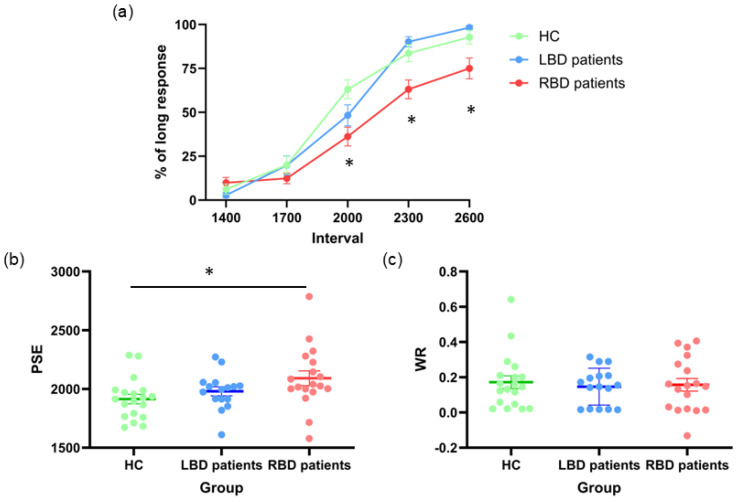
Time Estimation. (**a**) Mean values of long response (expressed in percentage %); (**b**) PSE values and (**c**) Weber Ratio (WR) for healthy controls (HC), left brain damaged (LBD) and right brain damaged (RBD) patients. Error bars indicate standard error means (SEM). Asterisk indicate significant differences.

The ANOVA on mean proportion of “long” response revealed a main effect of **Group** [F_(2,50)_ = 7.22, *p* = 0.002; η^2^_p_ = 0.22]. The post-hoc test highlighted a lower frequency of “long” responses in RBD patients (39%, 95% CI [0.34, 0.45]), if compared to both LBD patients (52%, 95% CI [0.46, 0.58]; *p* = 0.01) and HC (53%, 95% CI [0.48, 0.59]; *p* = 0.003), indicating time underestimation in RDB patients. No difference emerged between LBD patients and HC (*p* = 0.95). The factor **Interval** was significant [F_(4,200)_ = 271.33, *p* < 0.0001, η^2^_p_ = 0.84]: the percentage of “long” response increased as the time interval increased (1400 = 0.06, 1700 = 0.17, 2000 = 0.49, 2300 = 0.79 and 2600 = 0.88; all ps < 0.02). Interestingly, the interaction between Group*Interval was also significant [F_(8,200)_ = 4.56, *p* < 0.0001; η^2^_p_ = 0.15]: post-hoc analysis revealed that RBD patients underestimated as compared with HC on stimuli of 2000 ms (RBD: 36% vs. HC: 63%, *p* = 0.001), and as compared to LBD on stimuli of 2300 (RBD: 63% vs. LBD: 90%, *p* = 0.004) and 2600 ms (RBD: 75% vs. LBD: 98%, *p* = 0.03) (Figure 3a).

When considering PSE values as a dependent variable, the One-way ANOVA confirmed a significant effect of **Group** [F_(2,50)_ = 3.54; *p* = 0.04; η^2^_p_ = 0.12] (Figure 3b). Post-hoc analyses revealed that RBD patients had higher PSE values (mean= 2092 ms, 95% CI [1995, 2188]) with respect to HC (1914 ms, 95% CI [1820, 2008]; *p* = 0.03) but not LBD patients (1981 ms, 95% CI [1878, 2083]; *p* = 0.28). No difference emerged between LBD patients and HC (*p* = 0.63).

The One-way ANOVA on WR failed to reveal significant differences [Group: F_(2,50)_ = 0.14; *p* = 0.87; η^2^_p_ = 0.006], indicating that all groups equally modulated their responses depending on the differences between the standard and the comparison durations (HC = 0.17, 95% CI [0.11, 0.24]; LBD patients = 0.15, 95% CI [0.08, 0.22]; RBD patients = 0.16, 95% CI [0.09, 0.22]), which is indicative of comparable time sensitivity across the three groups (Figure 3c).

#### 3.1.2. Time Reproduction Computerized Task

**Figure 4:**
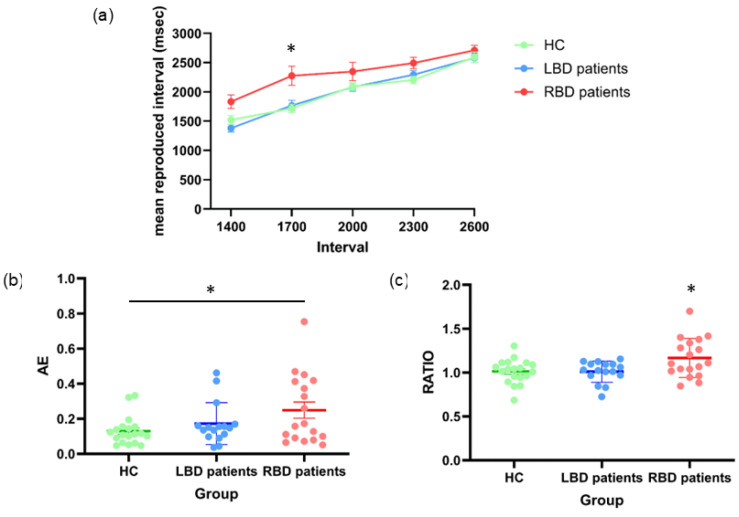
Time Reproduction. (**a**) Mean values of reproduced time intervals (expressed in msec); (**b**) Absolute Error (AE) and (**c**) RATIO for healthy controls (HC), left brain damaged (LBD) and right brain damaged (RBD) patients. Error bars indicate standard error means (SEM). Asterisks indicate significant differences.

The ANOVA on mean reproduced interval revealed a main effect of **Group** [F_(2,50)_ = 5.05, *p* = 0.01, η^2^_p_ = 0.17]. Post-hoc analyses showed that RBD patients reproduced longer time intervals (mean= 2332 ms; SD= 446 ms; 95% CI [2174, 2491]) as compared to both LBD patients (2021 ms, SD= 241 ms, 95% CI [1853, 2189]; *p* = 0.03) and HC (2027 ms, SD= 274 ms, 95% CI [1872, 2181]; *p* = 0.03). No difference emerged between LBD patients and HC (*p* = 0.99). The factor **Interval** was also significant [F_(4,200)_ = 124.26, *p* < 0.0001, η^2^_p_ = 0.71]: the reproduced time increased as the interval to-be-timed increased (1400 = 1585 ms, 1700 = 1924 ms, 2000 = 2175 ms, 2300 = 2330 ms and 2600 = 2630 ms; all ps < 0.02). Moreover, the interaction Group*Interval was significant [F_(8,200)_ = 2.60, *p* = 0.01, η^2^_p_ = 0.09]: stimuli lasting 1700 ms were reproduced longer in RBD patients (2277 ms) compared to HC (1719 ms; *p* = 0.007) and LBD patients (1769 ms; *p* = 0.04), whereas no difference was found comparing HC and LBD patients (*p* = 0.99). No significant difference emerged for other interval durations across groups (all ps > 0.12) (Figure 4a).

The One-way ANOVA conducted on AEs showed a significant effect of **Group** [F_(2,50)_ = 3.47, *p* = 0.04, η^2^_p_= 0.12]: post-hoc comparisons revealed that RBD patients (0.25, 95% CI [0.18, 0.32]) exhibited a worse performance compared to HC (0.13; 95% CI [0.07, 0.19]; *p* = 0.03) but not LBD patients (0.17; 95% CI [0.10, 0.24]; *p* = 0.27). No difference between HC and LBD patients was found (*p* = 0.66) (Figure 4b).

The One-way ANOVA considering RATIO as a dependent variable revealed a statistically significant effect of **Group** [F_(2,50)_ = 5.05, *p* = 0.01, η^2^_p_ = 0.17]. Post-hoc analysis showed that RBD patients significantly over-reproduced time (1.17, 95% CI [1.09, 1.25]) compared to HC (1.01, 95% CI [0.94, 1.09]; *p* = 0.02) and LBD patients (1.01, 95% CI [0.93, 1.09]; *p* = 0.03). No differences were found between HC and LBD patients (*p* = 0.99) (Figure 4c).

### 3.2. Experiment 2

#### 3.2.1. Time Estimation Virtual Reality Task

**Figure 5:**
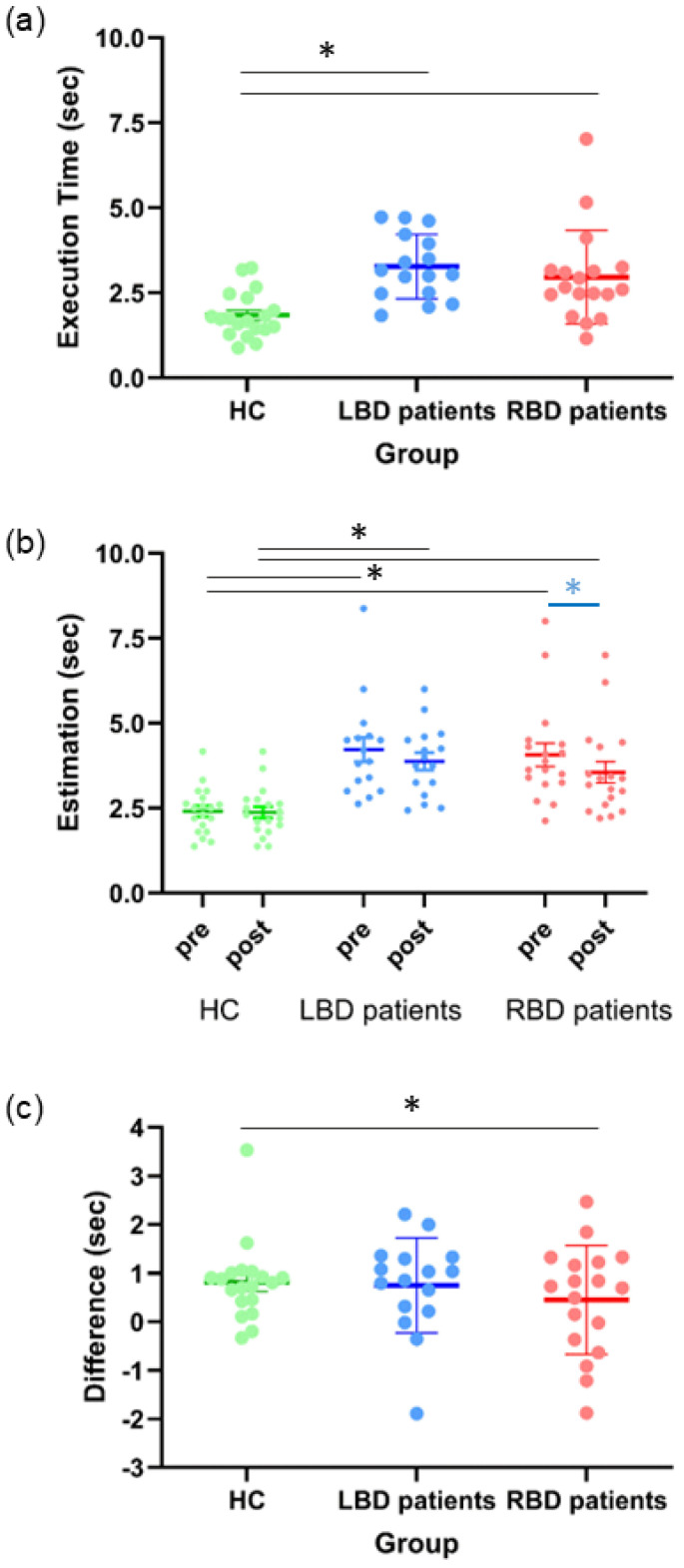
The graph indicates mean values of (**a**) Execution time (E_Est_), (**b**) Estimation (pre and post) and (**c**) the Difference (Estimation pre—Estimation post) for healthy controls (HC), left brain damaged (LBD) and right brain damaged (RBD) patients. Error bars indicate standard error means (SEM). Asterisks indicate significant within-group differences (blue) and between-group differences (grey).

The one-way ANOVA on E_Est_ revealed a main effect of **Group** [F_(2,50)_ = 9.49, *p* = 0.0003, η^2^_p_ = 0.28]: RBD (2.96, 95% CI [2.47, 3.45]) and LBD patients (3.28, 95% CI [2.76, 3.80]) were slower than HC (1.85, 95% CI [1.37, 2.32]; all ps < 0.006). No difference emerged between RBD and LBD (*p* = 0.67) (Figure 5a).

The ANOVA on Estimation scores revealed a main effect of **Group** [F_(2,50)_ = 11.94, *p* < 0.0001, η^2^_p_ = 0.32]: both RBD (3.81 s, 95% CI [3.29, 4.34]) and LBD patients (4.05 s, 95% CI [3.50, 4.60]) estimated significantly longer duration than HC (2.39 s, 95% CI [1.89, 2.90]; all ps < 0.001). No difference emerged between RBD and LBD patients (*p* = 0.82). Moreover, the factor **Session** [F_(1,50)_ = 14.10, *p* = 0.0004, η^2^_p_ = 0.22] and interaction Group*Session [F_(2,50)_ = 3.28, *p* = 0.0456, η^2^_p_ = 0.12] were also significant. Estimation post differed from Estimation pre and this was particularly true for RBD patients (post = 3.56 s, 95% CI [3.06, 4.05] vs. pre= 4.07 s, 95% CI [3.49, 4.65]; *p* = 0.005) who reduced their estimates after action execution compared to before. Conversely, Estimation post and Estimation pre were comparable both in HC (post = 2.38 s, 95% CI [1.90, 2.86] vs. pre = 2.41 s, 95% CI [1.85, 2.98]; *p* = 0.99) and in LBD patients (post = 3.88 s, 95% CI [3.35, 4.40] vs. pre = 4.22 s, 95% CI [3.61, 4.84]; *p* = 0.18) (Figure 5b).

The one-way ANOVA on Difference (Estimation pre—Estimation post) revealed a main effect of **Group** [F_(2,50)_ = 3.28, *p* = 0.0456, η^2^_p_ = 0.12]: RBD patients (0.51, 95% CI [0.24, 0.79]) had a greater difference between the two estimates as compared to HC (0.04, 95% CI [−0.23, 0.30]; *p* = 0.04). No significant difference emerged between RBD and LBD patients (0.35, 95% CI [0.06, 0.64]; *p* = 0.69), as well as between LBD and HC (*p* = 0.29) (Figure 5c).

#### 3.2.2. Time Reproduction Virtual Reality Task

The one-way ANOVA on E_Repr_ scores revealed a main effect of **Group** [F_(2,50)_ = 8.55, *p* = 0.0006, η^2^_p_ = 0.25]: RBD patients (3.80 s, 95% CI [3.20, 4.40]) were significantly slower than HC (2.08 s, 95% CI [1.50, 2.66]; *p* < 0.0006) in action execution. No difference emerged between RBD and LBD patients (2.89 s, 95% CI [2.26, 3.53]; *p* = 0.11), nor between HC and LBD patients (*p* = 0.18).

**Figure 6:**
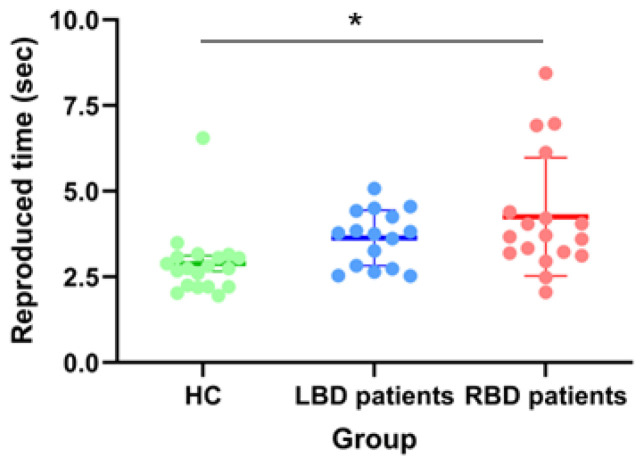
The graph indicates mean values (expressed in seconds) of Reproduced time scores for healthy controls (HC), left brain damaged (LBD) and right brain damaged (RBD) patients. Error bars indicate standard error means (SEM). Asterisks indicate significant differences.

The one-way ANOVA on mean Reproduced time revealed a main effect of **Group** [F_(2,50)_ = 5.51, *p* = 0.007, η^2^_p_ = 0.18]: RBD patients (4.25 s, 95% CI [3.66, 4.84]) reproduced longer than HC (2.89 s, 95% CI [2.31, 3.47]; *p* = 0.006). No difference emerged between HC and LBD patients (3.64 s, 95% CI [3.01, 4.26]; *p* = 0.22), neither between RBD and LBD patients (*p* = 0.35) (Figure 6).

### 3.3. Regression Analyses

The regression analyses conducted on *Time Estimation virtual reality task* revealed a significant effect of E_Est_ on Estimation scores: both temporal estimations provided before and after action execution were predicted by E_Est_ in HC (pre: r^2^ = 0.27; *β* = 0.52; *p* = 0.02; post: r^2^ = 0.40; *β* = 0.63; *p* = 0.004), LBD patients (pre: r^2^ = 0.31; *β* = 0.56; *p* = 0.02; post: r^2^ = 0.44; *β* = 0.66; *p* = 0.005) and RBD patients (pre: r^2^ = 0.25; *β* = 0.50; *p* = 0.04; post: r^2^ = 0.47; *β* = 0.69; *p* = 0.001).

The regression analysis conducted on *Time Reproduction virtual reality task* revealed a significant effect of E_Repr_ on Reproduction scores: temporal reproduction of actions was predicted by E_Repr_ in all groups (HC: r^2^ = 0.33; *β* = 0.58; *p* = 0.01; RBD: r^2^ = 0.64; *β* = 0.80; *p* = < 0.001; LBD: r^2^ = 0.33; *β* = 0.58; *p* = 0.02).

Conversely, the regression analyses conducted to investigate the relationship between computerized and VR tasks did not reveal any significance. Neither Estimation pre nor Estimation post in VR were predicted by the sensitivity index (WR) reported in the time estimation computerized task in all groups: HC (pre: r^2^ = 0.05; *β* = 0.22; *p* = 0.37; post: r^2^ = 0.09; *β* = 0.30; *p* = 0.22), RBD (pre: r^2^ = 0.005; *β* = 0.07; *p* = 0.77; post: r^2^ = 0.02; *β* = 0.13; *p* = 0.61), LBD (pre: r^2^ = 0.001; *β* = 0.04; *p* = 0.90; post: r^2^ = 0.06; *β* = 0.24; *p* = 0.37). Similarly, reproduction time in VR was not predicted by the AE observed in the time reproduction computerized task in any group (HC: r^2^ = 0.010; *β* = 0.10; *p* = 0.69; RBD: r^2^ = 0.02; *β* = 0.15; *p* = 0.55; LBD: r^2^ = 0.21; *β* = 0.46; *p* = 0.07).

Overall, this pattern of data suggests a dissociation between computerized and VR tasks.

### 3.4. Correlation Analyses

#### 3.4.1. Computerized Tasks

##### RBD Group

The Pearson correlation analysis between the proportion of long responses in the Time Estimation task and mean reproduction values in the Time Reproduction task revealed a significant negative correlation (r = −0.48; *p* = 0.04): the lower the proportion of long responses (time underestimation), the higher the mean values of reproduction (over-reproduction).

Considering the neuropsychological measures, a negative correlation was found between the PSE and spatial attentional abilities (BIT, r = −0.49; *p* = 0.046): a time underestimation is associated with a worse performance in spatial attentional abilities measured by the BIT battery. Moreover, a negative correlation was found between the time reproduction task and the time subscale of STEP (mean reproduction, r = −0.46 *p* = 0.05). No significant correlations were found considering the FIM (all ps > 0.88) and the FAB test (all ps > 0.15). Finally, the WR did not reveal any significant correlation with the measures of neuropsychological performance (all ps > 0.12).

##### LBD Group

The Pearson correlation analysis between the proportion of long responses in the Time Estimation task and mean reproduction values in the Time Reproduction task failed to reveal a significant correlation (r = −0.11; *p* = 0.68).

Considering the neuropsychological measures, the WR negatively correlated with executive functions (FAB, r = −0.60; *p* = 0.02): the higher the WR (i.e., poorer time sensitivity), the worse the executive performance. No significant correlations were found with other parameters (all ps > 0.27). The correlations for each group are reported in Table 4.

#### 3.4.2. Virtual Reality Tasks

##### RBD Group

A negative correlation was found between execution time in the time reproduction task and the motor subscale of FIM (r = −0.71; *p* = 0.05): the more the time needed to execute an action, the less the motor abilities. A negative correlation was also found between E_Est_ and the weight subscale of STEP (r = −0.50; *p* =.04): the more the time needed to execute an action, the worse the weight estimate.

##### LBD Group

Negative correlations between time subscale of STEP and all indices of estimation measured in VR (E_Est_, r = −0.68; Estimation pre, r = −0.63; Estimation post, r = −0.57; all ps < 0.02) were found. See Table 5 for details on correlation’s analysis.

### 3.5. Lesion Mapping

**Figure 7:**
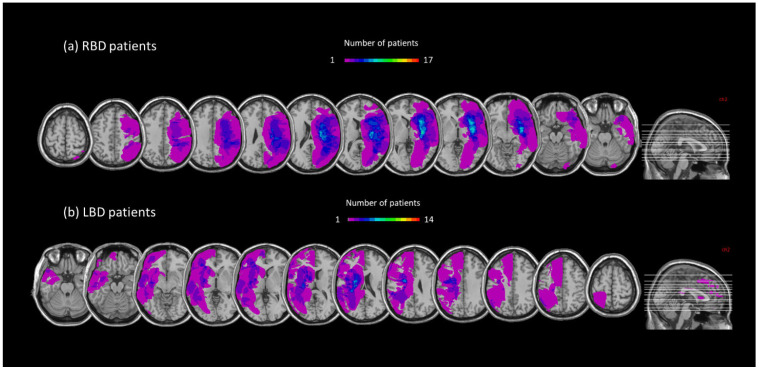
Overlay lesion plots are shown, separately for RBD ((**a**), upper panel) and LBD patients ((**b**), lower panel).

In RBD patients the area of maximal overlap of brain lesions covered the insula, putamen, caudate, superior corona radiata, external and internal capsule (Figure 7a). In LBD patients the area of maximal overlap covered the insula, putamen, caudate and pallidum (Figure 7b). A t- test performed over the lesion volume showed that the two groups did not differ in terms of lesion volume (t_29_ = −0.42; *p*= 0.68).

#### 3.5.1. Time Estimation Computerized Task

**Figure 8:**
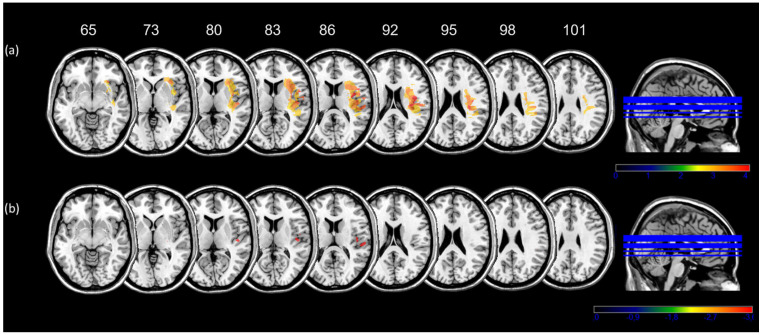
Brain regions significantly associated with time underestimation and over-reproduction. Panel (**a**) High z-scores (red) indicate that lesions to these voxels have a highly significant association with the underestimation of time intervals (Time Estimation computerized task). (**b**) High z-scores (red) indicate that lesions to these voxels have a highly significant association with the over-reproduction of time intervals (Time Reproduction computerized task). Only voxels that were significant at *p* = 0.05 are shown. Axial slices are numbered according to the Montreal Neurological Institute z coordinate.

VLSM showed that lesions involving a right cortico-subcortical fronto-parieto-temporal network were significantly associated with a temporal underestimation of interval in the Time estimation computerized task (Figure 8a). The threshold for statistical significance was z = 2.470 (corrected, *p* < 0.01) and the maximum voxels values were around right inferior frontal gyrus (35; 22; −5; z = 4.055, *p* < 0.01), the superior temporal gyrus (49; −26; 14; z = 3.34, *p* < 0.01), the precentral gyrus (49; 11; 11; z = 4.055, *p* < 0.01), the insula (37; −4; 12; z = 4.055, *p* < 0.01), the inferior parietal lobule (39; −25; 27; z = 3.34, *p* < 0.01), the anterior portion of the internal capsule (22; 9; 18; z = 3.41, *p* < 0.01), the external capsule (24; 7; 18; z = 3.41, *p* < 0.01) and corona radiata (25; 13; 18; z = 3.41, *p* < 0.01).

#### 3.5.2. Time Reproduction Computerized Task

VLSM showed that lesions involving a right parieto-temporal network were significantly associated with a temporal over-reproduction in the Time reproduction computerized task (Figure 8b). The threshold for statistical significance was z = −3.409 (corrected, *p* < 0.05) and the maximum voxels values were located in the superior temporal gyrus (59; −22; 13; z = −3.46, *p* < 0.05), the insula (45; −14; 11; z = −3.46, *p* < 0.05) and the postcentral gyrus (54; −26; 15; z = −3.46, *p* < 0.05).

#### 3.5.3. Time Estimation Virtual Reality Task

For each participant, we calculated the best-fitting linear equations relating the time estimated before and after action execution (Estimation pre and post) to the time executed. Then, the resultant slope parameters indicated how the time estimated changed (i.e., steepness) according to execution of actions. The Voxel-based lesion-symptom mapping on the slope parameters failed to reveal significant regions associated with performance in virtual reality.

#### 3.5.4. Time Reproduction Virtual Reality Task

**Figure 9:**
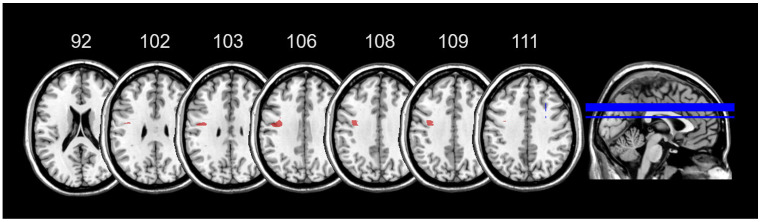
Brain regions significantly associated with slope parameters relating the time reproduced to the time executed in the Time Reproduction Virtual Reality task. Lesions to these voxels (red) were significant at *p* = 0.05. Axial slices are numbered according to the Montreal Neurological Institute z coordinate.

We calculated the best-fitting linear equations relating the time reproduced to the time executed for each participant. Then, the resultant slope parameters indicated how the time reproduced changed (i.e., steepness) according to the action’s execution. The VLSM on the slope parameters revealed that higher steepness in the time reproduction virtual reality task was significantly associated (threshold z = 3.23, corrected, *p* < 0.05) with lesion in the anterior portion of the left central sulcus (−37; −6; 34 z = 3.26, *p* < 0.05) encompassing premotor [BA 4: −45; −9; 32] and motor cortex [BA 6: −38; −3; 32] along the left superior longitudinal fasciculus (−42; −8; 31 z = 3.26, *p* < 0.05) (Figure 9).

## 4. Discussion

Time processing is a fundamental dimension of our everyday life. Research has shown that right-brain damaged patients show a deficit in computerized tasks that assess the time duration of visual stimuli, but do not explore whether this deficit also exists in timing daily living activities. To tackle this open issue, we adopted a well-known computerized task [6,22] and developed a novel virtual reality (VR) task that allowed us to judge the duration of one’s own actions framed in a realistic context in virtual reality scenarios. We expected an impairment in RBD, but not LBD, patients in the perception of duration of a visual stimulus [6,7,8,9,10,11,12], but this impairment could be attenuated in the Virtual Reality (VR) task. This assumption is mainly based on the idea that judging the duration of one’s own actions in the realistic context of VR might elicit motor imagery and mental simulation processes, which in turn facilitate temporal processing. To verify these hypotheses, patients with right and left brain damage, and a group of healthy controls, were submitted to two experiments by using a computerized and a VR paradigm, in which estimation and reproduction of temporal stimuli were required.

First of all, the results of the computerized tasks in Experiment 1 are in line with our predictions: RBD patients showed a concurrent time underestimation and over-reproduction as compared to healthy controls and LBD patients. Indeed, RBD patients systematically classified temporal intervals as shorter in the Time Estimation computerized task (i.e., a bias towards time underestimation) and reproduced them as longer in the Time Reproduction computerized task. According to the SET theory [29], we speculate that a right hemisphere lesion interferes with the alignment of real and perceived time: the perceived interval beats slower than the real one, inducing a slowdown of the encoding rate by the internal clock. Given that, the time underestimation and over-reproduction co-occurred in RBD patients: the same slowdown of the internal clock mechanism could have distorted the internal representations of time also while reproducing the encoded intervals, thus leading to a prolongation of their duration (i.e., an over-reproduction [6]). Thus, the shorter the time interval, the longer it will be reproduced. It is worth to note that the temporal deficit observed in RBD patients cannot be ascribed to a general difficulty in temporal discrimination because all participants correctly differentiated each temporal duration: indeed, their responses increased as the length of time interval increased. Moreover, when the Weber Ratio (WR) was considered, no difference emerged among the three groups, suggesting that time sensitivity, i.e., the ability to discriminate the length of intervals, was comparable in patients and controls.

The VLSM analysis revealed a distributed cortico-subcortical network located in the right hemisphere significantly associated with time underestimation: the inferior frontal gyrus, insula, caudate’s body, and a subcortical periventricular portion of the inferior parietal region. These results align with previous lesion studies on time underestimation [6,12] and recent meta-analyses on time processing both in healthy and neuropsychological populations [64,65]. Notably, focusing on the brain lesions associated with an over-reproduction of temporal intervals, we disclosed a subset of right cortical brain regions: insula, superior temporal and post-central gyri. Since these areas partially overlap with those associated with time underestimation, it is likely to suggest that an area that is involved in time reproduction is at least partially involved also in time estimation. Coherently, there was a significant correlation between patients’ performance at Time Estimation and Time Reproduction in RBD but not in LBD group. Together, these results seem to indicate that the temporal estimation mechanism is involved in temporal reproduction.

Undoubtedly, the involvement of the right inferior frontal gyrus points to the relevance of higher order cognitive processes, such as attention and working memory, to perform time estimation tasks, which are demanding in terms of cognitive resources [66]. The right inferior frontal gyrus has also been associated with the decision component of temporal estimation, especially when a comparison between two interval durations was required [29,33,34,67].

The right insular cortex is thought to integrate different signals originating from the outside world and within the individual, a function that is essential for building an interoceptive feeling of the state of the body moment after moment [68,69]. This integrative process would rely on an accumulation mechanism of physiological changes, which would be at the basis of our inner experience of time flowing [32,36,38]. Recent neuropsychological evidence by Teghil and colleagues [5] further demonstrated that the right insula is especially involved in timing when the context lacks informative cues and regularities to properly predict the stimulus duration.

Moving to the basal ganglia and the caudate nucleus, growing evidence demonstrated that these subcortical structures are intimately related to temporal processing thanks to their many connections with cortical regions. Accordingly, these structures would play the role of an “internal clock” capable of integrating the oscillatory cortical activity [65]. More specifically, the striatal beat frequency model proposed by Matell and Meck [70,71] suggests that synchronized frequencies of large cortical areas are the neural inputs constituting the temporal code for time representations.

Our results also disclosed a subcortical periventricular area within the right inferior temporo-parietal region significantly associated with time underestimation, and the lateral parietal cortex (post-central gyrus) with time over-reproduction. This is coherent with previous evidence on neurostimulation and neuropsychological studies in which, after a “virtual” or real lesion of the right temporo-parietal cortex, a bias in processing subsecond temporal intervals was found [72,73]. Together, these findings corroborate the idea that time processing relies on the functional interplay between temporal and parietal cortices in the right hemisphere, which act as a sensory-motor interface by connecting the central clock and peripheral motor effectors [74,75,76,77,78,79].

As regards the ability to judge the duration of one’s own actions framed by a realistic context in the VR scenarios, we hypothesize that participants can draw on the capacity to mentally simulate actions. Results of Experiment 2 showed that both RBD and LBD patients were impaired in time estimation of one’s own actions as compared to healthy controls. However, RBD but not LBD patients after action execution reduced the temporal estimates as compared to before action execution. This is in line with previous evidence showing that movement can enhance temporal perception and support a close link between action and interval timing [2,20,80,81,82]. This is striking since RBD patients exhibited a selective deficit when temporal estimation was assessed through the computerized task. Once they have performed the action, they can lock onto it, reducing the discrepancy between the duration of the performed action and its estimation. Regarding time reproduction, RBD patients did show a slowdown only compared to healthy controls, but not left brain damaged patients. Thus, RBD patients seem to especially benefit from action execution in a realistic context.

The overall pattern of results would suggest that the temporal impairment of RBD patients observed in computerized tasks was attenuated in VR tasks due to the possibility to rely on the mental simulation of the action, facilitated by the realistic context. Supporting this interpretation, regression analyses revealed that duration of action’s execution, but not indices of temporal performance, such as WR and AE, significantly predict both temporal estimation and reproduction measured in VR tasks. Consistently, correlation analyses between performance on temporal tasks and neuropsychological measures in RBD patients revealed that cognitive estimation abilities and attentional components were related to both estimation and reproduction of abstract durations (i.e., visual stimulus), whereas processing of sensory cues and motor-based competences were associated with the tasks in VR. Thus, if on one hand the recruitment of sensorimotor resources is primarily important for RBD patients, a different pattern of results was found in the LBD group. In fact, correlation analysis showed that the performance of these patients was mainly based on an abstract representation of the temporal interval. Accordingly, anatomo-lesional data revealed that a brain lesion encompassing left premotor and motor regions is selectively associated with a worse performance in reproducing the duration of actions in virtual reality scenarios. This finding is coherent with the involvement of frontal cortical regions dedicated to motor control (i.e., the supplementary motor area—SMA and pre-SMA) in motor timing tasks [1,5,65,83]. Furthermore, previous studies have shown that motor imagery activates a set of frontal motor areas, parietal areas, and cerebellar regions that partially overlap with the brain network that is activated during motor execution [83,84,85,86,87,88]. Interestingly, neuroimaging studies reported that motor imagery of different effectors activates the corresponding sections of the somatotopically organized motor cortex, supporting a close link between motor imagery and motor execution [89,90]. This is also coherent with evidence provided by Gavazzi and colleagues [81], who demonstrated an improvement of participants’ temporal accuracy only when the kinematics of visual stimuli is congruent with the internal model of action within the human compared to non-human motor repertoire. Strikingly, previous evidence coming from a fMRI study by Ferri and colleagues [91] on bodily self-recognition showed that left motor and premotor cortices, among others, are engaged when participants performed a mental rotation of self-body parts (i.e., hand). This adds support to our hypothesis that mental simulation processes (i.e., motor imagery) that recruit the motor areas of the left hemisphere are involved in processing the temporal duration of actions in virtual reality tasks. Therefore, patients whose brain areas required for the mental simulation of actions are spared may take advantage of mentally imagining actions when they judge their temporal duration. This process is facilitated by VR scenarios that reproduce typical, familiar and concrete everyday contexts for patients. If this is the case, our findings may help in clarifying why temporal disorders are more often revealed in the clinical setting, whereas they are rarely reported by patients in the ecological setting. To date, indeed, most of the studies have investigated temporal deficits through computerized tasks [6,7,8,9,10,11,12], which are not fully representative of timing in everyday life activities. This is confirmed by the regression analyses showing that the temporal performance in “abstract” computerized tasks does not predict the “concrete” virtual tasks. Therefore, exploring time deficits in a more ecological, though controlled way is of undoubted importance, and the current study enables us to take a first step in this direction.

## 5. Limitations

The present findings are promising, although some limitations should be mentioned. First of all, the current study focuses exclusively on motor aspects, but it is well known that a proprioceptive component also plays a role in movement, which, however, has not been investigated in this work. Second, temporal disorders are more often revealed in a clinical context, whereas they are rarely reported by patients in an ecological context. Along this line, Magnani and colleagues described a patient whose temporal deficit, investigated through a qualitative interview, negatively impacts daily routines [92]. Thus, it is possible that in our study, the computerized task failed to capture all nuances of the disorder, such as the subjective aspects of temporal difficulties.

## 6. Conclusions and Future Directions

The current findings suggest that the temporal deficits typically seen in RBD patients during computerized tasks (i.e., underestimation and over-reproduction of temporal durations) were less pronounced when they judged the duration of their own actions in virtual reality settings, likely because the realistic context of VR supported mental action simulation.

Since VR seems to be the best candidate for exploring temporal deficits in a more ecological context (see for a review, [93,94,95]), future studies should explore time processing in RBD patients with neglect, whose temporal deficit is greater than in RBD patients without neglect [7,12]. Indeed, as revealed by Magnani and colleagues [92], temporal deficit seems to severely impact everyday life activities in neglect patients. Thus, for clinical and rehabilitative purposes [96], it is crucial to investigate whether these patients may or may not take advantage of mentally simulating an action in everyday ecologically valid contexts recreated through VR. Addressing this issue could lead to more effective and personalized intervention strategies for clinical populations.

## Figures and Tables

**Table 1:** Demographic and Neuropsychological data for left (LBD) and right brain damaged (RBD) patients.

Case	Aetiology	TPL	MMSE	Token Test	BIT-C	BCT		AT		FAB	STEP	
						OM	Left OM	Full Apples	Asymmetry		Time	Weight
LBD 1	H	45	28.5	26.8	145	0	1	50	0	14.5	17 *	11 *
LBD 2	I	2	26.7	NA	NA	NA	NA	NA	NA	NA	22	26
LBD 3	I	25	30	33.5	145	0	0	47	−2	14.8	27	20
LBD 4	H	12	19 *	NA	139	5	0	50	0	11.5 *	NA	NA
LBD 5	I	1	26	33.5	144	NA	NA	47	−1	16.1	22	24
LBD 6	I	2	26	31.5	134	6 *	3	45	1	12.4 *	21	28
LBD 7	I	30	27.7	33	140	1	1	50	0	13.5	22	23
LBD 8	I	8	20.3 *	27.8	143	1	1	50	0	NA	25	18 *
LBD 9	H	10	30	34.3	143	0	0	49	0	16.1	27	23
LBD 10	TBI	5	26.2	30	NA	0	0	49	−1	16.2	26	14 *
LBD 11	I	1	27	35.3	140	1	0	48	−1	15.8	24	24
LBD 12	I	1	29.3	33.3	145	3	1	45	2	16.4	22	25
LBD 13	I	1	26.3	32	144	6 *	2	50	0	13.2 *	24	24
LBD 14	I	67	28.7	32.5	143	1	1	49	−1	12.5 *	24	24
LBD 15	I	4	26	25.8 *	143	2	0	49	1	14.4	22	26
LBD 16	H	4	24	32.8	140	5	3	50	0	15.4	21	24
RBD 1	H	37	27	33.3	143	2	1	50	0	14.1	28	26
RBD 2	I	1	24.7	30.3	140	4	2	41 *	−1	11.9 *	17 *	18 *
RBD 3	I	16	25.9	30	143	1	1	49	1	17.7	25	24
RBD 4	I	82	28	35.8	144	0	0	48	−1	13.2 *	23	25
RBD 5	I	5	25.3	35	138	1	1	46	−2	15.3	19 *	28
RBD 6	I	1	30	NA	NA	2	0	48	1	NA	25	25
RBD 7	I	145	25.2	32.5	138	1	0	50	0	13.7	24	21
RBD 8	I	1	21.1 *	30.5	137	0	0	49	1	12.5 *	26	24
RBD 9	H	16	25.3	27.3	139	4	4	48	0	13.3 *	22	27
RBD 10	H	117	27	34.2	138	1	1	49	1	10.9 *	25	23
RBD 11	I	10	27	31.5	146	0	0	50	0	16.9	18 *	28
RBD 12	I + H	34	27.2	32.5	136	3	0	46	0	13.2 *	23	17 *
RBD 13	I	90	26	33	137	1	0	50	0	12.9 *	16 *	21
RBD 14	I	18	25.2	31.8	144	0	0	50	0	15.9	19 *	24
RBD 15	I	61	27	34.3	137	4	4	48	2	14.1	21	20
RBD 16	N	1	26.2	31.5	142	8 *	5 *	47	0	15.9	23	23
RBD 17	H	63	25.4	28	130	4	1	46	2	15.7	20	18
RBD 18	I	7	26.2	30	120 *	2	1	45	−1	16.7	22	17 *

Aetiology: H = Hemorrhage; I = Ischemia; TBI = Traumatic Brain Injury; N = Neoplasia; TPL = Time Post Lesion is indicated in months; MMSE = Mini Mental State Examination (cut off > 24); Token Test (cut off > 26.5); BIT-C = Behavioral Inattention Test—Conventional subtest (cut off > 129); BCT = Bells Cancellation Test: number of total omissions (OM; cut off < 5) and left omissions are reported (left OM; cut off < 5); AT = Apple Test number of full apples barrage (cut off > 45) and full apple asymmetry (cut off < 2); FAB = Frontal Assessment Battery (cut off > 13.5); STEP = Time and Weight Estimation Test (cut off > 20); NA = not available. Asterisk indicates a pathological performance.

**Table 2:** Demographic and Clinical data for healthy controls (HC).

Case	Sex	MMSE	STEP	
			Time	Weight
HC 1	F	26.2	25	21
HC 2	F	26.2	26	23
HC 3	F	26.4	25	21
HC 4	F	26.4	25	16 *
HC 5	M	30	24	13 *
HC 6	M	30	25	25
HC 7	F	30	28	23
HC 8	M	30	22	22
HC 9	F	30	23	22
HC 10	F	28.3	24	23
HC 11	M	27	23	20
HC 12	F	26.2	22	26
HC 13	F	26.7	24	15 *
HC 14	F	26.2	22	25
HC 15	M	23.2	23	19 *
HC 16	F	30	21	20
HC 17	F	25.2	27	24
HC 18	F	30	23	25
HC 19	F	25.2	22	27

Sex (F = female; M = male); Age is indicated in years; MMSE = Mini Mental State Examination (cut off > 24); STEP = Time and Weight Estimation Test (cut off > 20). Asterisk indicates a performance below the cut-off.

**Table 3:** Mean scores (bold) and Standard deviation (italic) of Estimation and Reproduction VR tasks for healthy controls (HC), left brain damaged (LBD) and right brain damaged (RBD) patients.

	Execution Time of Estimation	Estimation Pre	Estimation Post	Execution Time of Reproduction	Reproduction
HC	**1.845**	**2.414**	**2.379**	**2.079**	**2.889**
	*0.664*	*0.686*	*0.713*	*0.509*	*0.989*
LBD	**3.276**	**4.225**	**3.878**	**2.891**	**3.636**
	*0.951*	*1.432*	*1.035*	*1.183*	*0.809*
RBD	**2.965**	**4.072**	**3.558**	**3.802**	**4.252**
	*1.373*	*1.449*	*1.318*	*1.792*	*1.727*

**Table 4:** Coefficient r (bold) and *p* value (italic) of correlations between computerized tasks and neuropsychological performance for right brain damaged (RBD) and left brain damaged (LBD) patients.

		STEP Time	STEP Weight	FIM	FAB	BIT−C
**RBD**	PSE	**0.0284**	**−0.1918**	**0.0709**	**0.2602**	**−0.4900**
		*0.911*	*0.446*	*0.868*	*0.313*	*0.046*
	WR	**−0.1590**	**0.0902**	**−0.3521**	**−0.3943**	**0.3159**
		*0.529*	*0.722*	*0.392*	*0.117*	*0.217*
	Mean reproduction	**−0.4646**	**−0.0857**	**0.134**	**0.3636**	**0.0871**
		*0.05*	*0.735*	*0.752*	*0.151*	*0.74*
**LBD**	PSE	**0.1463**	**−0.0694**	**0.1863**	**−0.0863**	**−0.0238**
		*0.603*	*0.806*	*0.631*	*0.769*	*0.936*
	WR	**−0.2410**	**0.0911**	**−0.2301**	**−0.5971**	**−0.3095**
		*0.387*	*0.747*	*0.551*	*0.024*	*0.282*
	Mean reproduction	**−0.3023**	**0.0486**	**−0.1382**	**0.1411**	**0.0586**
		*0.273*	*0.863*	*0.723*	*0.63*	*0.842*

**Table 5:** Coefficient r (bold) and *p* value (italic) of correlations between VR tasks and neuropsychological performance for right brain damaged (RBD) and left brain damaged (LBD) patients.

		STEP Time	STEP Weight	FIM	FAB	BIT−C
**RBD**	E_Est_	**−0.0880**	**−0.4955**	**−0.4281**	**−0.0111**	**−0.1945**
		*0.728*	*0.037*	*0.29*	*0.966*	*0.454*
	Estimation pre	**0.0775**	**−0.0407**	**−0.5936**	**0.0028**	0.1344
		*0.76*	*0.873*	*0.121*	*0.991*	*0.607*
	Estimation post	**0.0367**	**−0.2274**	**−0.5600**	**0.1075**	**−0.0180**
		*0.885*	*0.364*	*0.149*	*0.681*	*0.945*
	E_Repr_	**−0.1018**	**−0.4402**	**−0.7084**	**−0.2527**	**−0.1850**
		*0.688*	*0.068*	*0.049*	*0.328*	*0.477*
	Reproduction	**−0.2274**	**−0.4400**	*−0.594*	*−0.1541*	*−0.2847*
		*0.364*	*0.068*	*0.121*	*0.555*	*0.268*
**LBD**	E_Est_	**−0.6821**	**0.1538**	**−0.4104**	**−0.4670**	**−0.2212**
		*0.005*	*0.584*	*0.273*	*0.092*	*0.447*
	Estimation pre	**−0.6262**	**0.1885**	**−0.4402**	**−0.1418**	**−0.3817**
		*0.013*	*0.501*	*0.236*	*0.629*	*0.178*
	Estimation post	**−0.5763**	**0.2443**	**−0.1384**	**−0.2241**	**−0.2061**
		*0.025*	*0.38*	*0.723*	*0.441*	*0.48*
	E_Repr_	**−0.3783**	**−0.1131**	**−0.0097**	**−0.4257**	**−0.3007**
		*0.164*	*0.688*	*0.98*	*0.129*	*0.296*
	Reproduction	**−0.4068**	**0.0987**	**−0.0425**	**−0.4524**	**−0.0520**
		*0.132*	*0.726*	*0.914*	*0.104*	*0.86*

## Data Availability

All relevant data and materials from our study will be made available by the authors upon request.

## Supplementary Materials

The following supporting information can be downloaded at: https://www.mdpi.com/article/10.3390/jcm14144825/s1.
